# Application of olefin metathesis in the synthesis of functionalized polyhedral oligomeric silsesquioxanes (POSS) and POSS-containing polymeric materials

**DOI:** 10.3762/bjoc.15.28

**Published:** 2019-02-04

**Authors:** Patrycja Żak, Cezary Pietraszuk

**Affiliations:** 1 Adam Mickiewicz University in Poznań, Faculty of Chemistry, Umultowska 89b, 61-614 Poznań, Poland

**Keywords:** olefin metathesis, POSS, silsesquioxanes

## Abstract

This mini-review summarizes the applications of olefin metathesis in synthesis and functionalization of polyhedral oligomeric silsesquioxanes (POSS) and POSS-containing polymeric materials. Three types of processes, i.e., cross metathesis (CM) of vinyl-substituted POSS with terminal olefins, acyclic diene metathesis (ADMET) copolymerization of divinyl-substituted POSS with α,ω-dienes and ring-opening metathesis polymerization (ROMP) of POSS-substituted norbornene (or other ROMP susceptible cycloolefins) are discussed. Emphasis was put on the synthetic and catalytic aspects rather than on the properties and applications of synthesized materials.

## Introduction

**Figure 1:**
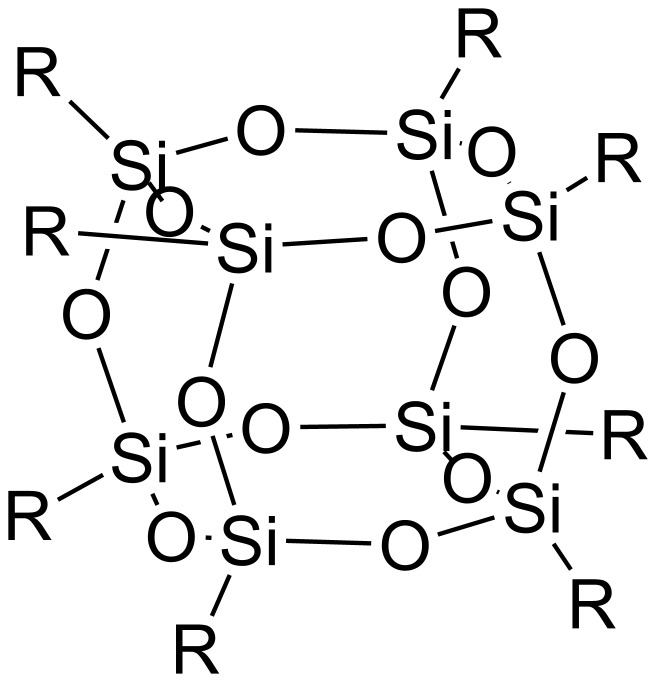
Cubic octasilsesquioxane.

Silsesquioxanes are nanostructures described by the empirical formula RSiO_3/2_, where R represents hydrogen, alkyl, alkenyl, aryl, arylene or their functionalized derivatives. A number of silsesquioxane structures have been reported including random, ladder, cage and partial cage structures. Silsesquioxanes with specific cage structures are commonly referred as polyhedral oligomeric silsesquioxanes (POSS). From among POSS structures the most thoroughly studied is a cubic silsesquioxane unit, denoted also as **T_8_**. It contains an inorganic cubic core composed of eight Si atoms at the vertices, connected through O atoms along the edges, chemically bonded with eight different or similar organic substituents so that it represents a truly hybrid architecture. The cubic silsesquioxane unit is characterized by a three-dimensional nanoscopic size structure with approximate Si–Si distance equal to 0.5 nm and an approximate R–R distance of 1.5 nm (Figure 1). The synthesis, structure and properties of POSS have been extensively reviewed [1–3].

Proper selection of organic substituents R allows the modification of solubility of POSS in reaction media, its compatibility with polymers, biological systems, or surfaces. The introduction of one or more reactive groups into the POSS structure permits their further chemical modification. Because of the ease of the synthesis as well as the commercial availability of polyhedral oligomeric silsesquioxanes containing vinyl groups (which is a common functional group used in organosilicon chemistry), POSS are often functionalized through the chemical processes of C=C bond transformation, e.g., hydrosilylation, Heck coupling, silylative coupling and olefin metathesis.

Olefin metathesis, i.e., catalytic exchange of double bonds between carbon atoms, is a powerful tool in organic synthesis. The use of metathesis in organic and polymer synthesis is comprehensively described in excellent monographs [4–6]. However, the literature does not offer a more detailed review on the application of metathesis in the synthesis of functionalized polyhedral oligomeric silsesquioxanes (POSS). The lack of a pertinent overview in this field has prompted us to summarize the reported applications of olefin metathesis in the synthesis and functionalization of oligomeric silsesquioxanes and POSS-containing polymeric materials. This review is focused on the synthetic and catalytic aspects rather than on the properties and applications of the resulting materials.

**Scheme 1:**
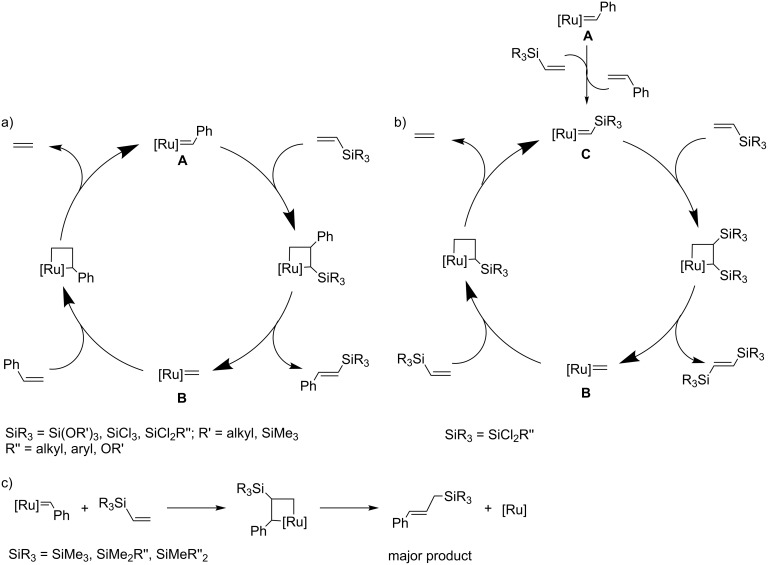
Reactivity of vinylsilanes in the presence of ruthenium alkylidene complexes; a) cross metathesis, b) homometathesis, and c) decomposition of β-silylruthenacyclobutane.

Vinylsilanes show a specific reactivity towards alkylidene ruthenium complexes because of a strong effect of the silyl group on the properties of the double bond. In general, the substituents at the silicon atom determine the regioselectivity of the vinylsilane cycloaddition to the Ru=C bond. The knowledge of this untypical reactivity is pivotal for the application of metathesis for the modification of vinylsilanes, vinyl-substituted siloxanes, spherosilicates and silsesquioxanes. The appropriate choice of substituents permits the control of the process to a certain degree. The reactivity of vinylsilanes with different substituents at silicon towards alkylidene ruthenium complexes is illustrated in Scheme 1 [7].

According to Scheme 1a, as a result of the reaction of trialkoxy-, tris(trimethylsiloxy)-, trichloro- or dichloromethyl-substituted vinylsilanes with Grubbs catalyst of first or second generation (**A**), the active methylene complex **B** and the corresponding (*E*)-1-phenyl-2-(silyl)ethene are formed. The methylene complex **B** in the presence of styrene undergoes metathetic conversion to benzylidene complex **A** and ethene. When dichloro-substituted vinylsilanes are used, the pathway shown in Scheme 1b is also possible. Metathesis of dichloro-substituted vinylsilanes with Grubbs catalyst **A** leads to styrene and (silyl)methylidene complex **C**. Formation of (silyl)methylidene complex **C** has not been confirmed by spectroscopic methods. The reaction of the postulated complex **C** with vinylsilane gives the corresponding (*E*)-1,2-bis(silyl)ethenes and the methylene complex **B**. The methylene complex **B** may react with vinylsilane to form ethene and regenerate complex **C**. In the presence of vinylsilanes containing alkyl substituents the Grubbs catalyst undergoes fast decomposition as a result of β-transfer of the silyl group in the appropriate β-(silyl)rutenacyclobutane complex to ruthenium followed by reductive elimination of the corresponding propene derivative (Scheme 1c). The transformation resulted in complexes that do not contain a carbene ligand and do not show catalytic activity in metathesis.

**Figure 2:**
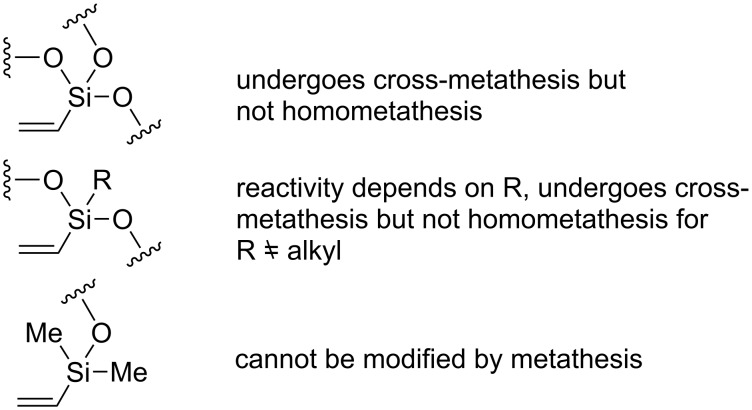
The scope and limitations of metathesis in transformations of vinyl-substituted siloxanes and silsesquioxanes.

The most important consequences of the above-described reactivity in metathesis of vinyl-substituted siloxanes, spherosilicates and silsesquioxanes are presented in Figure 2. It should be indicated that one of the consequences of the described reactivity is the inactivity of vinylsilsesquioxane in homometathesis.

The limitations apply to silanes containing a double bond located directly at the silyl group and do not apply to allylsilanes and other alkenylsilanes, which behave like terminal olefins and readily undergo metathesis.

**Scheme 2:**
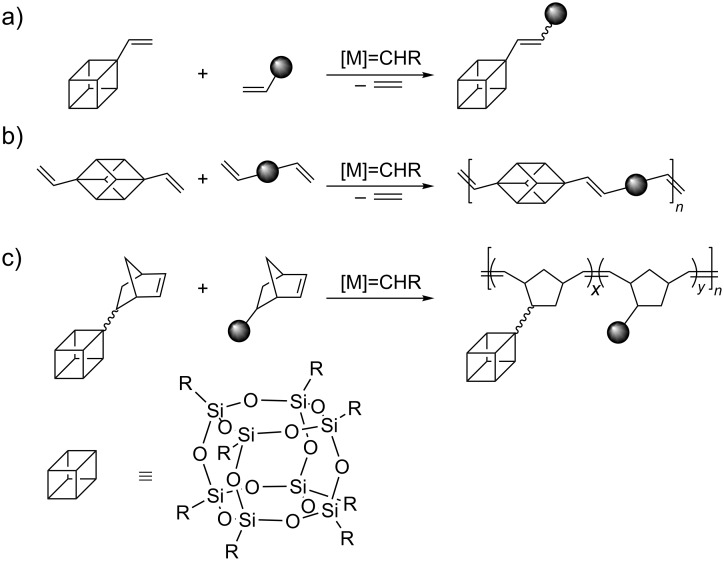
Application of olefin metathesis in the synthesis and modification of POSS-based materials: a) functionalization of vinyl-substituted POSS via cross metathesis; b) synthesis of POSS-containing polymers via acyclic diene metathesis; c) synthesis of POSS-containing copolymers via ROMP.

Application of metathesis in chemistry of unsaturated derivatives of POSS is limited to three types of processes, i.e., cross metathesis (CM) of vinyl-substituted POSS with terminal olefins, acyclic diene metathesis (ADMET) copolymerization of divinyl-substituted POSS with α,ω-dienes and ring-opening metathesis polymerization (ROMP) of POSS-substituted norbornene (or other ROMP susceptible cycloolefins, Scheme 2).

**Figure 3:**
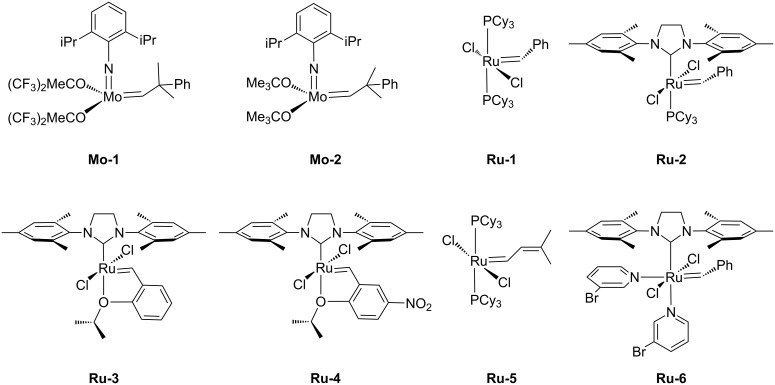
Olefin metathesis catalysts used in transformations of silsesquioxanes.

Nearly all metathetic transformations described in this review have been performed in the presence of commonly used ruthenium-based catalysts (Figure 3). In contrast, there are only a few examples of application of molybdenum-based complexes in modification of silsesquioxanes (Figure 3), which can be explained as related to the sensitivity of these complexes toward atmospheric oxygen, moisture and functional groups of reagents.

## Review

### Cross metathesis of vinyl-substituted silsesquioxanes

**Figure 4:**
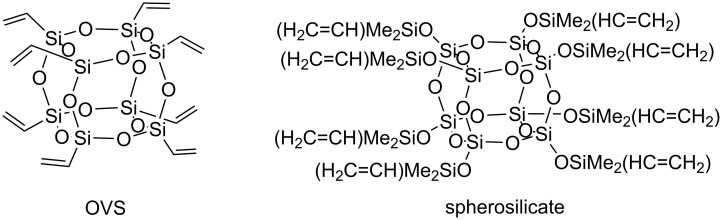
Octavinyl-substituted cubic silsesquioxane (OVS) and spherosilicate.

The first metathetic transformations of vinyl-substituted silsesquioxanes and spherosilicates (Figure 4) were reported by Feher in 1997 [8]. In the presence of molybdenum alkylidene complex **Mo-1** octavinylsilsesquioxane (OVS) underwent cross metathesis of terminal and internal olefins, functionalized olefins (such as allyltrimethoxysilane, ethyl undec-10-enylate, oct-7-enyltrimethoxysilane, 5-bromopentene, pent-4-en-1-ol) and styrene.

Moreover, the catalytic activity of the first generation Grubbs’ catalyst (**Ru-1**) was demonstrated in CM of OVS with pent-4-en-1-ol and 5-bromopentene. It has been found that terminal alkenes undergo cross metathesis much more readily and are clearly better than internal alkenes from the cost perspective. However, internal alkenes are less volatile and cannot produce any ethene, which makes them interesting starting materials. A slight vacuum had to be applied to reactions with terminal alkenes in order to remove ethene, because ethene would strongly slow down the desired cross metathesis and inactivate Schrock-type metathesis catalysts. CM of OVS with styrenes proceeded stereoselectively. A mixture of *cis-* and *trans*-isomers was obtained in the transformations of other olefins tested. Spherosilicate was shown to undergo CM with pent-1-ene and styrene in the presence of **Mo-1**. No data on the activity of **Ru-1** in metathesis transformation of spherosilicates was provided.

**Scheme 3:**
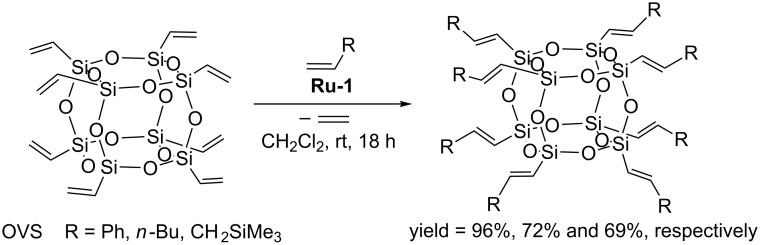
Cross metathesis of OVS with terminal olefins (stereoselectivity as discussed in the text).

In 2004 Marciniec reported the first efficient cross metathesis of octavinylsilsesquioxane (OVS) occurring in the presence of first generation Grubbs’ catalyst (**Ru-1**, Scheme 3) [9].

Octavinylsilsesquioxane (OVS) has been effectively transformed via cross metathesis with styrene, 1-hexene and allyltrimethylsilane. The reactions were carried out in the presence of first-generation Grubbs catalyst at room temperature using a 12- or 24-fold molar excess of olefin relative to silsesquioxane. The reaction with styrene led to the formation of the expected product with an exclusive *E*-stereochemistry around the newly formed C=C double bond, while aliphatic α-alkenes (1-hexene, allyltrimethylsilane) gave a mixture of stereoisomers (*E*/*Z* = 94:6). Additionally, when 1-hexene was used as reacting partner, the product of the cross metathesis was accompanied by considerable amounts of those of olefin homometathesis. Under optimized conditions, CM of OVS with styrene proceeds quantitatively despite the low loading of the catalyst (0.5 mol % relative to the vinylsilyl group, Scheme 3) [9]. Effective cross metathesis was observed when OVS was treated with vinyl sulfide in the presence of second generation Grubbs’ catalyst (**Ru-2**). The product was obtained in 91% isolated yield, however, the process required a temperature elevation to 60 °C and the use of a catalyst amount of 4 mol % [9].

**Scheme 4:**
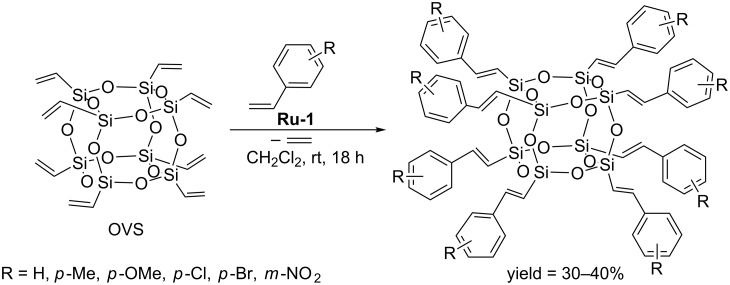
Cross metathesis of OVS with substituted styrenes.

Laine has described the cross metathesis of OVS with a series of substituted styrenes (Scheme 4) [10].

**Scheme 5:**
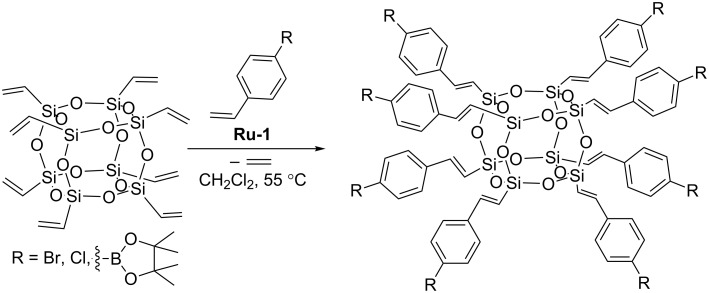
Modification of OVS via CM with styrenes.

Cross metathesis was carried out using a 1.5-fold excess of commercially available functionalized styrenes and 0.5 mol % of **Ru-1**. The reaction mixtures were stirred for 72 h to ensure complete conversion of the silsesquioxane. The quantitative conversion of the substrate can be achieved by blowing a gentle stream of nitrogen above the reaction mixture to remove the ethylene formed. The resulting 4-bromostyryl derivatives were subsequently modified via Heck coupling with a set of 4-substituted styrenes to give the next generation of functionalized derivatives. The authors also demonstrated the possibility of further functionalization of an amino-substituted derivative via the reaction with 3,5-dibromo or dinitrobenzoyl chloride. The proposed synthetic method based on the gradual development of the organic part can be used for the synthesis of new star polymers, dendrimers or hyperbranched molecules. Further examples of the use of cross metathesis of OVS with styrenes in order to form functionalizable dendrimer cores have been reported by Cole-Hamilton [11]. Procedures allowing the syntheses of POSS derivatives with synthetically useful functional groups in multigram quantities have been proposed (Scheme 5).

**Figure 5:**
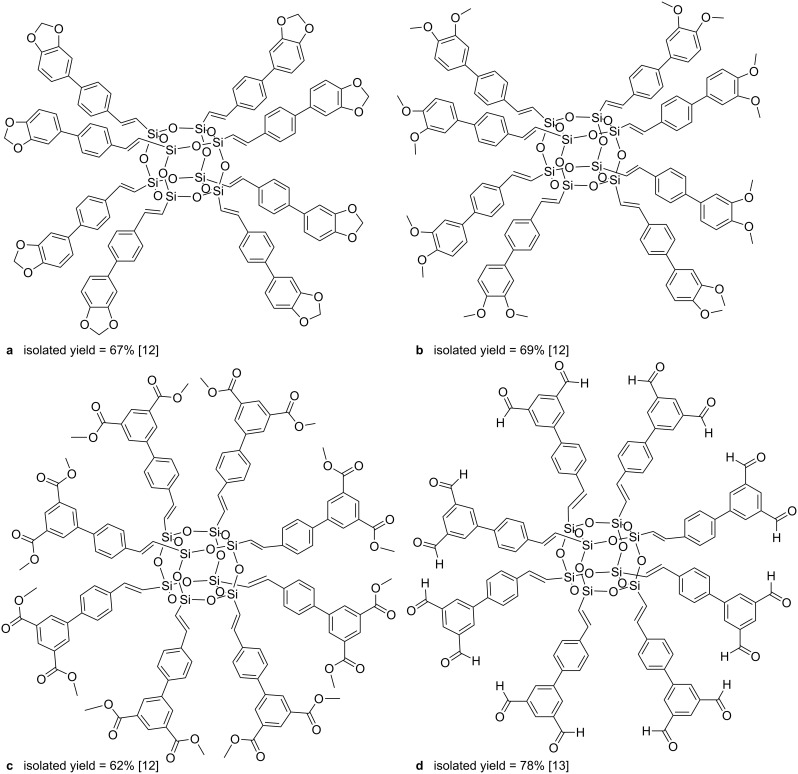
Vinylbiphenyl chromophore-decorated cubic silsesquioxanes.

A similar procedure permits the synthesis of a series of vinylbiphenyl chromophore-decorated cubic oligosilsesquioxanes [12–13]. In the process conditions applied (methylene chloride at 55 °C, **Ru-1**) cross metathesis has been accompanied by competitive olefin homometathesis. The authors have developed a method for the isolation and purification of the expected materials and obtained the desired derivatives (Figure 5) with isolated yields exceeding 60%.

Chromophore-functionalized silsesquioxane-core dendrimers were obtained to investigate their photophysical properties [12,14]. In the synthesized compounds chromophore properties were only slightly influenced by the core. The possibility of fine-tuning of the photophysical properties of the POSS-based dendritic molecule not only by changing the chromophore but also by providing tailored steric interactions between bridges and/or chromophores was proved [14]. Interestingly, the 4’-vinylbiphenyl-3,5-dicarbaldehyde group modified macromolecule (Figure 5d) displayed the ability to become luminescent when exposed to reducing agents such as NaBH_4_, LiAlH_4_ or BH_3_ [13].

Procedures for high yield and selective modification of octavinylsilsesquioxane (OVS) via CM with a variety of substituted styrenes, including the ones bearing highly π-conjugated substituents such as phenyl, 1-naphthyl, 9-anthracenyl and 2-thienyl have been reported by Marciniec [15]. For all styrene derivatives tested, the procedures described permitted highly regioselective metathesis leading to exclusive formation of the *E*-isomer. Cross-metathesis experiments were performed under mild reaction conditions (CH_2_Cl_2_, 40 °C, 24 h), in the presence of the first generation Grubbs catalyst (**Ru-1**). Under such conditions, a fully selective course of the reaction was observed.

**Scheme 6:**
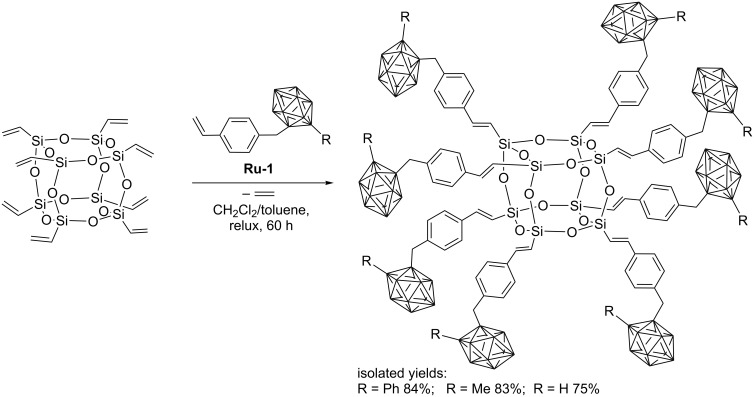
Cross metathesis of OVS with carboranylstyrene.

Núñez has described the synthesis of fluorescent POSS derivatives with carboranylstyrene fragments attached to each corner. The procedure involves CM of OVS with carboranylstyrene compounds with different substituents (Ph, Me, or H, Scheme 6) [16].

**Scheme 7:**
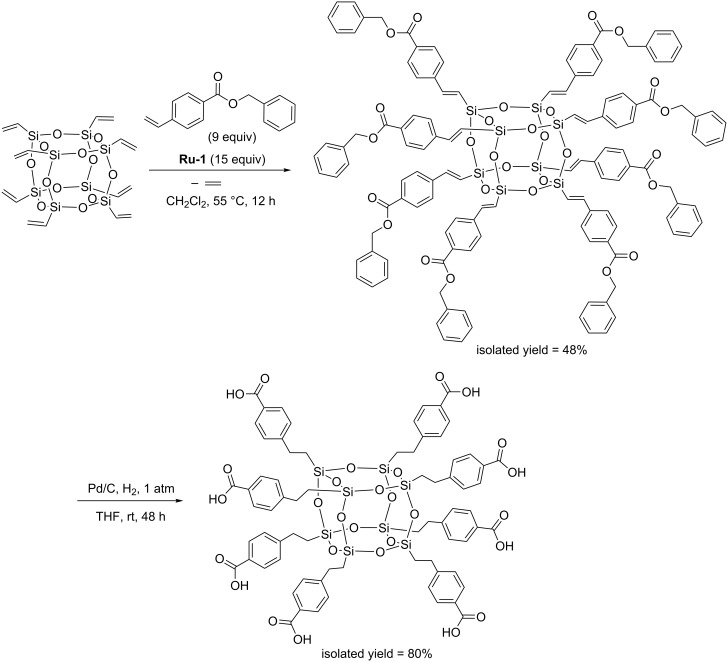
Synthesis of octakis[2-(*p*-carboxyphenyl)ethyl]silsesquioxane via CM and subsequent hydrogenation.

The reactions catalyzed by **Ru-1**, occurred with quantitative conversion and excellent regio- and stereoselectivity leading to exclusive formation of *E*-isomers. However, CM was accompanied by a minor amount of homometathesis. Fortunately, the product of homocoupling could be easily separated from the desired CM products. The presence of the carborane clusters was shown to enhance the thermal stability of the materials. Absorption and emission data of carborane–POSS hybrids indicate a large red-shift with respect to the precursors. Dautel and Moreau have synthesized octakis[2-(*p*-carboxyphenyl)ethyl] (Scheme 7) and octakis[2-(4-carboxy-1,1’-biphenyl)ethyl]silsesquioxane via cross-metathesis methodology [17]. In the presence of palladium and dihydrogen the synthesized derivatives undergo, under mild conditions, hydrogenolysis of the benzyl ester group to the carboxylic acid and hydrogenation of the C=C double bonds at the silicon atoms (Scheme 7). The ability of the obtained derivatives, in particular the carboxylic acids, to generate nanostructured materials through self-organization processes was tested. The X-ray crystal structures of the octaester showed an interpenetrated compact packing of the molecular building blocks without any specific supramolecular interaction. The structure of the octaacid was found to contain hydrogen-bonded ribbons, thanks to the two-dimensional character of the acid and the directionality of the hydrogen bond pattern of the acid dimer.

**Scheme 8:**
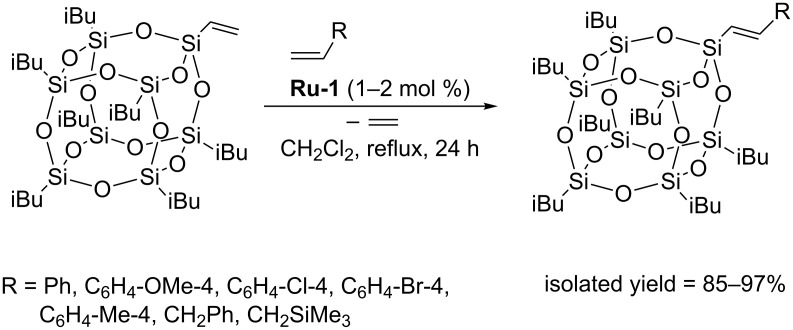
Cross metathesis of monovinyl-POSS with olefins.

Cross metathesis of monovinyl-substituted POSS with olefins has been reported for the first time by Marciniec [18]. It was demonstrated that monovinylheptaisobutyl-substituted octasilsesquioxane (monovinyl-POSS) underwent highly efficient CM with styrenes as well as vinyl and allyl organic derivatives in the presence of **Ru-1** (Scheme 8).

**Scheme 9:**
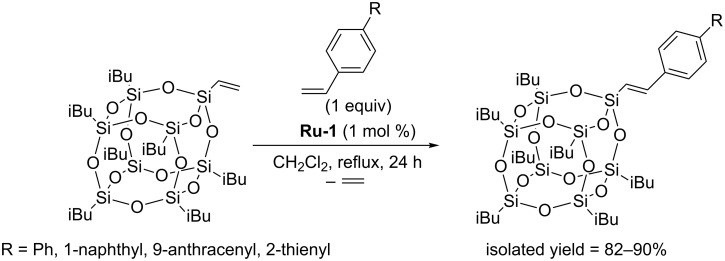
Cross metathesis of monovinyl-POSS with highly π-conjugated substituted styrenes.

The reactions were performed in refluxing methylene chloride in the presence of usually 1 mol % of first generation Grubbs catalyst (**Ru-1**) and led to the formation of the expected products with isolated yields ranging from 85% to 97%. In all cases the exclusive formation of *E*-isomers was detected and the formation of competitive olefin homometathesis was not observed. The reactions were carried out using a small excess of olefin (1.5–3 equiv) to ensure complete conversion of the reactants. In the reaction of monovinyl-POSS with allylbenzene, CM was accompanied by double bond migration, which results in reduction of the isolated yield of the CM product (85%) and the formation of minor amounts (15%) of 1-propenylbenzene. No similar isomerization was observed in the reaction of POSS with allyltrimethylsilane. Further research enabled Marciniec to extend the scope of the reaction by reporting efficient CM of monovinyl-POSS with a series of substituted styrenes. The reported procedures permit efficient and selective functionalization of mono- and octavinylsilsesquioxanes with π-conjugated substituents via cross metathesis (Scheme 9) [15].

**Scheme 10:**
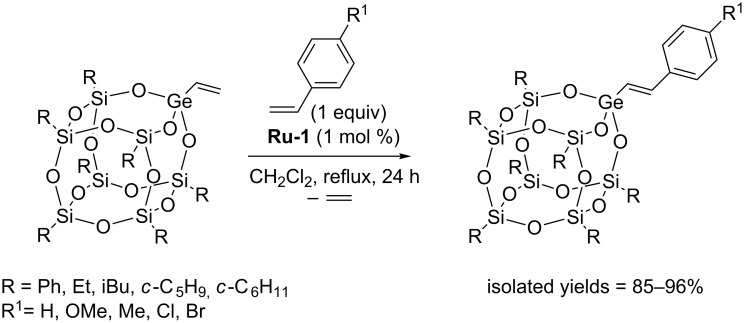
Cross metathesis of monovinylgermasilsesquioxane with styrenes.

In 2016 Marciniec reported the synthesis of a series of new cubic POSS in which one vertex silicon atom was replaced by a germanium atom bearing a vinyl group [19]. Monovinylgermasilsesquioxanes were successfully converted into the corresponding styryl derivatives via CM with styrenes (Scheme 10). Under optimized reaction conditions complete conversion of reacting partners and selective formation of CM products with exclusive *E*-arrangement around the C=C double bonds was observed.

The most suitable catalyst for CM was found to be **Ru-1**, in whose presence no undesirable competitive reaction of olefin homometathesis occurred. Full conversion of monovinylgermasilsesquioxane required the use of 1 mol % of the catalyst. The reactions described are the first examples of metathesis activity of vinylgermanium compounds.

**Scheme 11:**
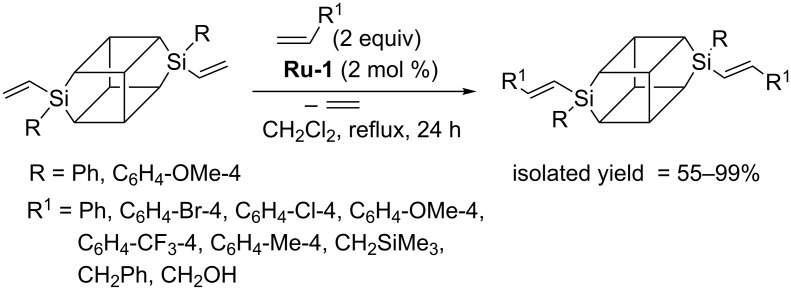
Cross metathesis of DDSQ-2SiVi with olefins.

More than a decade ago Yoshida developed a new class of silsesquioxyl compounds containing rigid Si–O–Si bonds, called double-decker silsesquioxanes [20–21]. This class of compounds has recently been reviewed [22]. Marciniec found that divinyl-substituted double-decker silsesquioxanes (DDSQ-2SiVi) can be functionalized via cross metathesis and provided a series of examples of effective CM of DDSQ-2SiVi with styrenes and selected allyl derivatives (Scheme 11) [23].

**Scheme 12:**
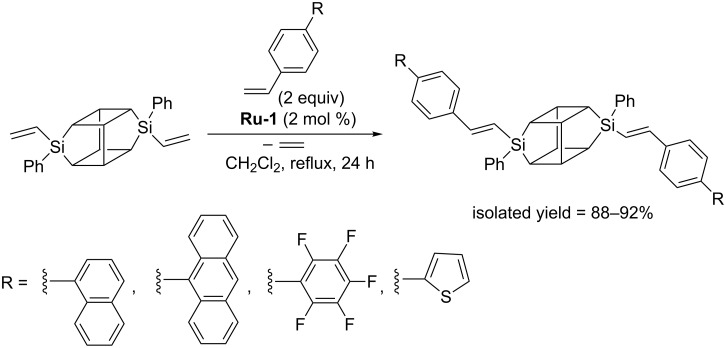
Cross metathesis of DDSQ-2SiVi with substituted styrenes.

Under optimized reaction conditions (Scheme 11), CM led to the exclusive formation of *E*-isomers and was not accompanied by competitive homometathesis. This selectivity was obtained thanks to the use of the **Ru-1** catalyst, moderately active in homometathesis of the olefins studied. Effective transformation was observed for substituted styrenes. Expected products were isolated with yields in the range of 88–95%. When allyl derivatives (allyltrimethylsilane, allylbenzene and allyl alcohol) were tested as olefinic partners, incomplete conversions of reactants (55–60%) were observed, despite the increased catalyst loading (2 mol %). Effective metathesis transformation was observed also in the presence of **Ru-2** but then considerable amounts of olefin homometathesis product were formed. The presence of a methyl group at the vinylsilyl moiety was responsible for the lack of activity, which was consistent with earlier studies (Scheme 1). The scope of the reaction was further extended to the palette of olefins containing conjugated systems of π-bonds (Scheme 12) [24].

**Scheme 13:**
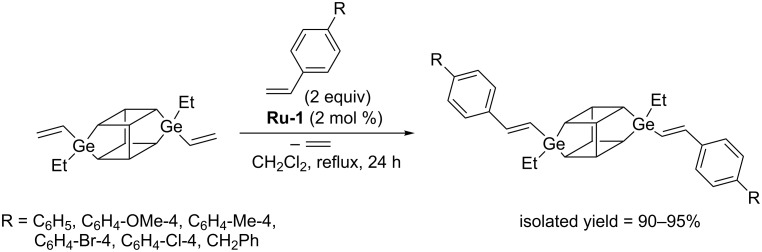
Cross metathesis of (DDSQ-2GeVi) with olefins.

Irrespective of the type of olefin used, under optimized conditions all reactions proceeded with high yields and stereoselectivity, leading to exclusive formation of the *E*,*E*-isomer. Marciniec reported the synthesis of divinylgermasilsesquioxane (DDSQ-2GeVi) and proved effective functionalization of such compounds by cross metathesis with a series of 4-substituted styrenes and allylbenzene, in the presence of **Ru-1** (Scheme 13) [19].

Under optimized conditions reactions led to fully chemo- and stereoselective formation of disubstituted germasilsesquioxanes. The ability of alkyldisiloxyvinylgermane to be converted in metathesis is worth noting as the analogous vinylsilane does not undergo metathesis.

**Scheme 14:**
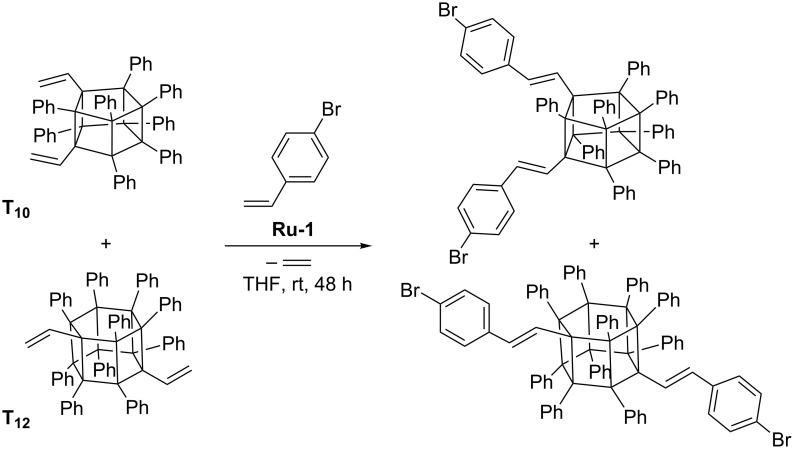
CM of divinyl-substituted **T_10_** and **T_12_** with 4-bromostyrene (selected isomers are shown).

In 2010 Laine reported a procedure enabling the synthesis of polyhedral vinylphenyl-substituted deca- and dodecasilsesquioxanes (denoted **T_10_** and **T_12_**, respectively) [25]. Divinyl octa- or decaphenylsubstituted **T_10_** and **T_12_** derivatives (mixture of isomers) were demonstrated to effectively undergo cross metathesis with 4-bromostyrene in the presence of **Ru-1** (Scheme 14).

**Scheme 15:**
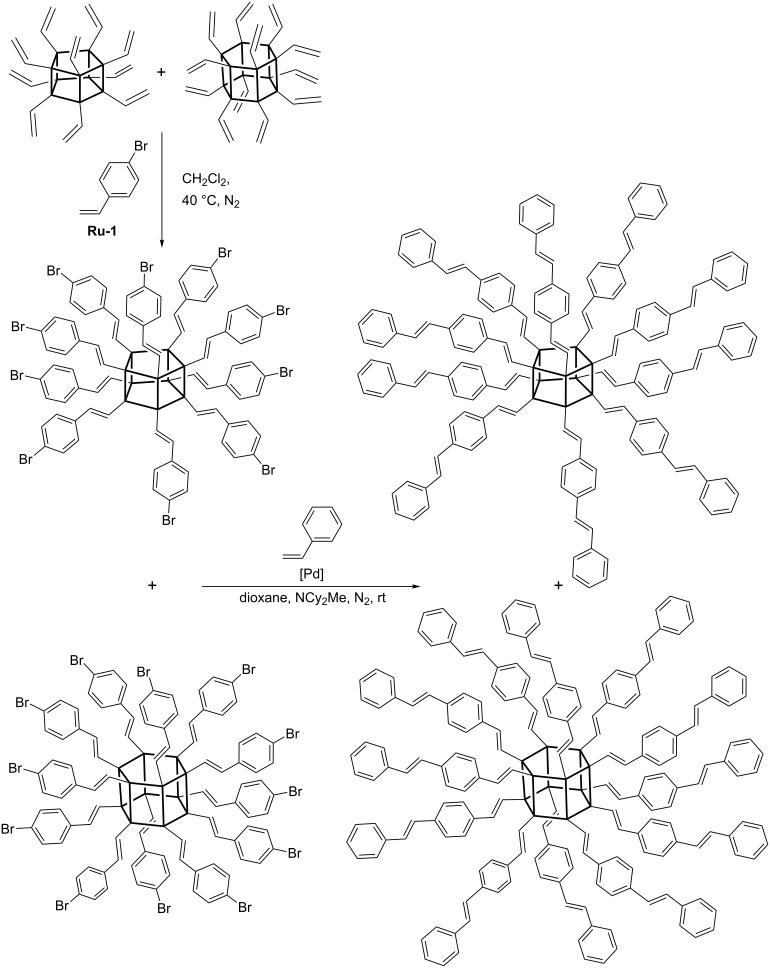
Synthesis of vinylstilbene derivatives of **T_10_** and **T_12_** via a sequence of CM and Heck coupling.

Attempts of homometathesis of vinylsilsesquioxanes have failed, which is understandable in view of the above presented scheme of reactivities of vinylsilanes (Scheme 1). The possibility to modify vinyl and styryl derivatives of silsesquioxanes via Heck reaction has been proved. The Heck coupling of 4-bromostyrene and vinyl-POSS derivatives leads to the formation of oligomeric products containing a silsesquioxane core in the polymer backbone. The deca- and dodecavinyl derivatives of **T_10_** and **T_12_**, respectively, undergo cross metathesis with 4-bromostyrene in the presence of **Ru-1** to form 4-bromostyryl derivatives, which in turn can be modified by Heck coupling with styrene to produce stilbenevinyl derivatives (Scheme 15) [26]. Laine has proposed a procedure for the separation of **T_10_** and **T_12_** derivatives, which enabled detailed photophysical studies of pure **T_10_** and **T_12_** core-based materials [26].

Detailed photophysical studies of chromophore-functionalized **T_10_** and **T_12_** silsesquioxanes have shown that the cage size and/or the symmetry can strongly affect photophysical properties [26]. In the subsequent paper the authors describe the use of OVS or mixtures of **T_10_** and **T_12_** units in the synthesis of hydroxyphenyl-terminated silsesquioxanes. Such derivatives were obtained via cross metathesis with 4-acetoxystyrene or via a sequence of cross metathesis with 4-bromostyrene and Heck coupling with 4-acetoxystyrene. The resulting acetoxy compounds were then hydrolyzed to produce hydroxy-functionalized derivatives. These compounds, after purification, were reacted with adipic acid chloride to form POSS-moiety containing highly crosslinked polyesters with some porosity [27].

**Scheme 16:**
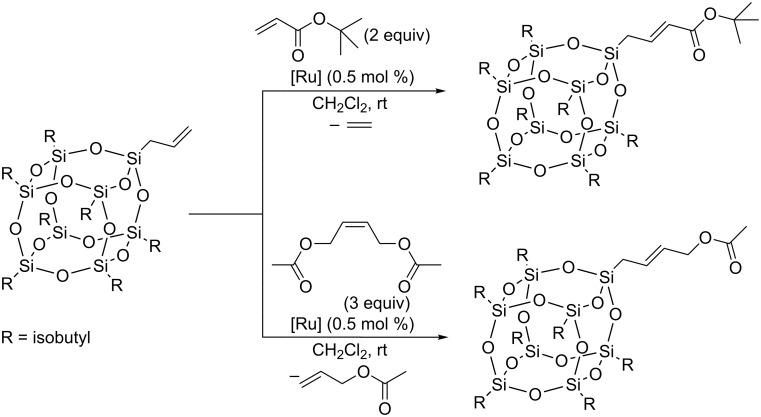
Cross metathesis of allyl-POSS with *tert*-butyl acrylate and (*Z*)-1,4-diacetoxy-but-2-ene.

Czaban-Jóźwiak and Grela have studied the metathetic transformation of allyl-substituted cubic silsesquioxane [28]. In search for the optimum catalyst a variety of ruthenium complexes were tested in the CM of allylsilsesquioxane with *tert*-butyl acrylate and (*Z*)-1,4-diacetoxybut-2-ene as model olefins (Scheme 16).

**Scheme 17:**
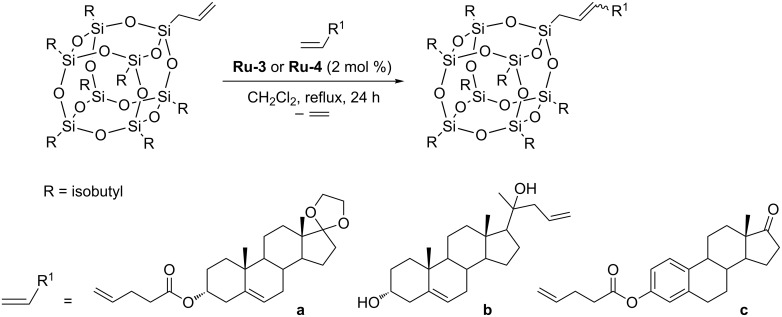
Cross metathesis of allyl-POSS with olefins.

For the majority of the ruthenium catalysts tested, despite the mild reaction conditions, high yields were observed. No reaction or lower yields of the test reaction products were observed for first generation catalysts and indenylidene complexes. For further research, active in preliminary tests and commercially available second generation Grubbs–Hoveyda catalyst **Ru-3** and its nitro derivative **Ru-4** were selected. The same authors were able to successfully functionalize allylsilsesquioxane with more challenging, three different steroid derivatives. The reactions were performed in toluene at 100 °C in the presence of 2 mol % of **Ru-3** or **Ru-4** (Scheme 17, substrate **a** or in CH_2_Cl_2_ at 45 °C in the presence of 2 mol % of **Ru-4** (Scheme 17, substrates **b** and **c**). The products were obtained with yields of 62–72% as a mixture of *Z*/*E* isomers in the ratio of 20:80. Efficient homometathesis of allylsilsesquioxane occurring in toluene at 100 °C in the presence of 0.5 mol % of **Ru-4** was noted. The observed activity of allylsilsesquioxane in homometathesis is understandable because allylsilanes (unlike vinylsilanes) behave in metathesis like terminal olefins.

**Scheme 18:**
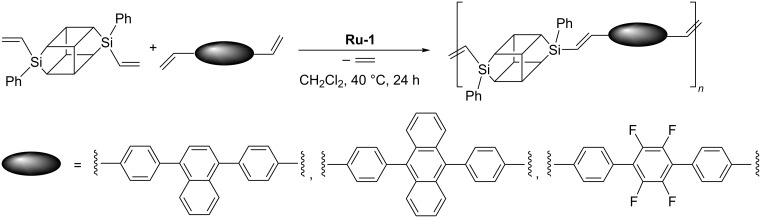
Acyclic diene metathesis copolymerization of DDSQ-2SiVi with diolefins.

There are scarce reports on the application of ADMET in the synthesis of oligomers or polymers containing a POSS unit in the main chain. Marciniec disclosed ADMET copolymerization of DDSQ-2SiVi with dienes in the stereoselective synthesis of a new class of vinylene–arylene copolymers containing double-decker silsesquioxanes in the main chain (Scheme 18) [24].

**Scheme 19:**
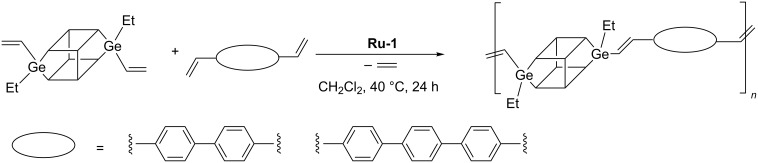
Acyclic diene metathesis copolymerization of DDSQ-2GeVi with diolefins.

The products were polymers characterized by *M*_n_ in the range from 9100 to 18300 Da and *M*_w_ in the range from 13600 to 46100 Da. Thermogravimetric analyses indicate a high level of thermal resistance of the obtained systems, reaching the temperature values over 550 °C. Analogous ADMET copolymerization of divinylgermasilsesquioxanes with 4,4'-divinylbiphenyl or 4,4''-divinylterphenyl can be used in the synthesis of stereoregular *trans*-germasilsesquioxyl–vinylene–phenylene oligomers (Scheme 19) [19].

This method permitted obtaining a polymer with *M*_w_ in the range from 9057 to 11033 Da and polydispersity index (PDI) = 1.5.

### Ring-opening metathesis polymerization (ROMP) of POSS-functionalized monomers

The chemistry of inorganic–organic hybrid materials has emerged as a fascinating new field of modern nanotechnology. The inclusion of POSS cages into the polymeric material can significantly improve such properties of the polymer as thermal and oxidative resistance, surface properties, improvement of mechanical properties as well as reduced flammability, heat release and viscosity during processing [29]. Synthesis, properties and applications of POSS-containing materials are the subject of numerous reviews [30–37]. From among the methods for preparation of organic–inorganic hybrid materials, polymerization or copolymerization is particularly convenient to incorporate POSS units into polyolefins.

Ring-opening metathesis polymerization (ROMP) is the type of olefin metathesis chain-growth polymerization that uses metathesis catalysts to generate polymers from cyclic olefins [38–41]. To obtain polymers functionalized with POSS in the side chain, a susceptible to the ROMP monomer connected via a suitable linker to the silsesquioxane cage should be used. Due to the ease of polymerization and functionalization, norbornene derivatives are the most often used monomers.

The aim of this section is to indicate the applications of ROMP in the synthesis of hybrid materials containing the POSS moiety covalently bonded to organic polymeric chains rather than the discussion of the properties of the obtained materials.

The synthesis of polymers by ROMP is carried out almost exclusively in the presence of ruthenium-based catalysts **Ru-1**–**Ru-6** because of their tolerance to moisture, atmospheric oxygen and most functional groups as well as commercial availability. The choice of solvents is determined by the solubility of monomers, with methylene chloride, chloroform, and toluene being the most commonly used. Polymerization is terminated by addition of ethyl vinyl ether to the reaction mixture. Ruthenium residues from the obtained copolymer are removed on a short alumina plug.

**Scheme 20:**
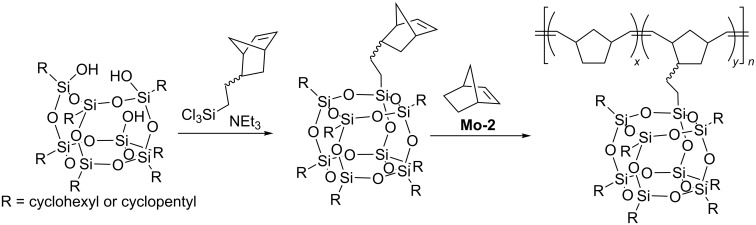
Ring-opening metathesis copolymerization of norbornenylethyl-POSS with norbornene.

In 1999 Lichtenhan reported ring-opening metathesis copolymerization of POSS-functionalized norbornene with norbornene in the presence of the Mo-based catalyst **Mo-2** (Figure 3, Scheme 20) [42]. The polymerization was carried out in CHCl_3_ under nitrogen atmosphere. The reactions were terminated by the addition of benzaldehyde. A series of random copolymers with different weight percentage of POSS containing comonomer were synthesized.

**Scheme 21:**
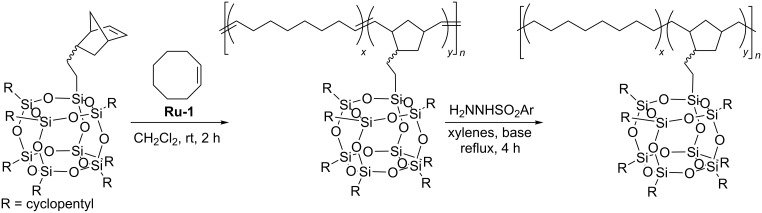
Synthesis of a polyethylene–POSS copolymer via ring-opening metathesis copolymerization of norbornenylethyl-POSS with cyclooctene and subsequent hydrogenation.

Ruthenium alkylidene catalyst **Ru-1** was successfully used by Caughlin who reported ring-opening metathesis polymerization of heptacyclopentylnorbornenylethyloctasilsesquioxane and its copolymerization with cyclooctene [43]. The obtained copolymer was subsequently hydrogenated to afford polyethylene–POSS random copolymer (Scheme 21). Thermogravimetric analysis of the polyethylene–POSS copolymers under air showed a significant improvement of the thermal stability relative to that of polyethylene.

**Scheme 22:**
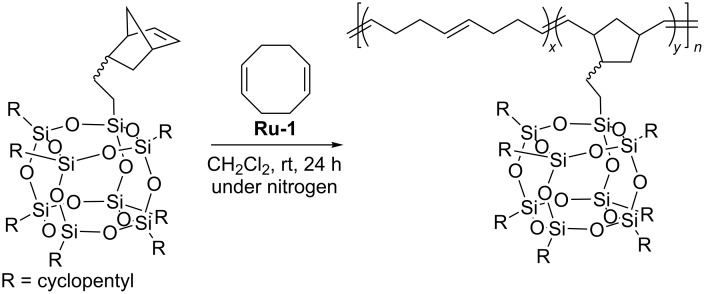
ROMP of norbornenylethyl-POSS with 1,5-cyclooctadiene.

In subsequent studies Caughlin used ring-opening metathesis copolymerization of POSS-functionalized norbornene with 1,5-cyclooctadiene in the presence of **Ru-1** for the synthesis of a series of random copolymers in which POSS loading varied in the range from 0 to 53 wt % (Scheme 22) [44]. Polymers with a weight-average molecular mass in the range from 67000 to 88000 Da were obtained.

**Scheme 23:**
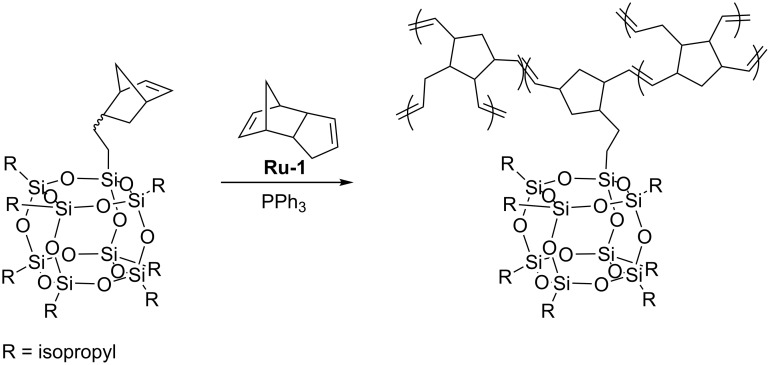
Copolymerization of POSS-functionalized norbornene with DCPD.

In the random copolymers obtained, the associative interactions between the particles were shown to result in the formation of ordered nanostructures. TEM micrographs indicate that the copolymers assemble into small, randomly oriented lamellae with lateral dimensions of approximately 50 nm and a thickness of ca 3–5 nm that corresponds to twice the diameter of a POSS nanoparticle. With increasing POSS concentration, the nanostructures extend to longer continuous lamellae having lateral lengths in the order of microns. Ruthenium alkylidene catalyst **Ru-5** was successfully used in copolymerization of cubic silsesquioxane bearing four β-styryl and four (3-phenyloxiran-2-yl) substituents with dicyclopentadiene (DCPD) [45]. Moreover, octanorbornenyl cubic silsesquioxane was found to undergo ring-opening metathesis copolymerization with DCPD. Due to limited solubility only 0.1 mol % of POSS was used in the copolymerization. Such a small content of the POSS-containing comonomer caused, however, an increase in *T*_g_ up to 15 °C in relation to that of polyDCPD. Similar examinations were reported by Coughlin who used first generation Grubbs catalyst (**Ru-1**) for copolymerization of POSS-functionalized norbornene with DCPD (Scheme 23) [46]. During polymerization, PPh_3_ had to be added to reduce the activity of **Ru-1**.

**Scheme 24:**
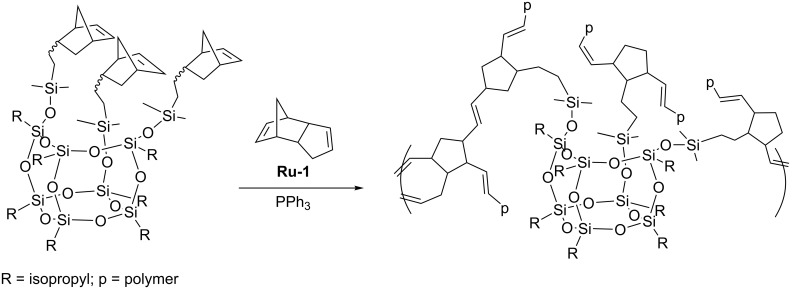
Copolymerization of tris(norbornenylethyl)-POSS with DCPD.

Dicyclopentadiene and norbornenylethyl-POSS or tris(norbornenylethyl)-POSS (Scheme 24) have been copolymerized over a range of POSS loadings. In the copolymers obtained using mononorbornylethyl-POSS, the aggregates containing three to four POSS molecules were observed for high POSS loadings. When tris(norbornenylethyl)-POSS was used as comonomer, the POSS remained uniformly dispersed over all loadings. No improvements in thermal properties were observed in the copolymers obtained.

**Scheme 25:**
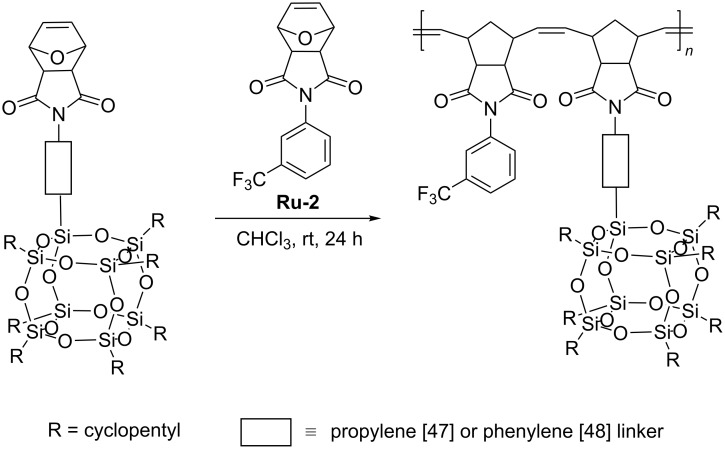
Copolymerization of *N*-(propyl-POSS)-7-oxanorbornene-5,6-dicarboximide with 3-(trifluoromethyl)phenyl-7-oxanorbornene-5,6-dicarboximide DCPD.

Another POSS-containing monomer – *N*-(propyl-POSS)-7-oxanorbornene-5,6-dicarboximide was tested in ring-opening metathesis copolymerization with 3-(trifluoromethyl)phenyl-7-oxanorbornene-5,6-dicarboximide in the presence of second generation Grubbs catalyst (**Ru-2**, Scheme 25) [47].

**Figure 6:**
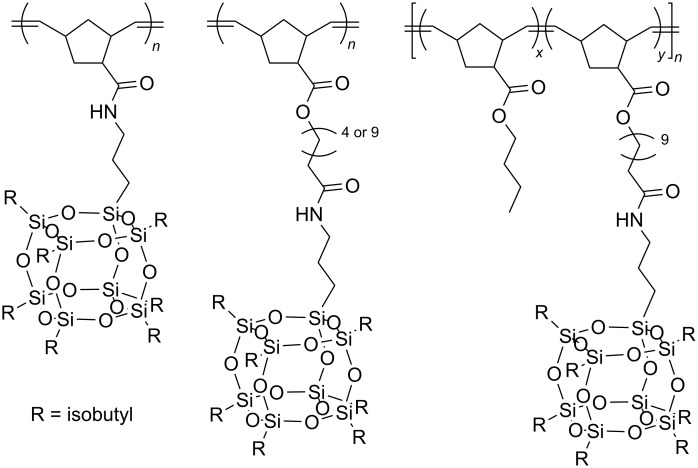
Homopolymers and copolymers having POSS groups attached to the main chain via flexible spacers of different lengths.

The use of specified proportions of the two comonomers allowed obtaining a series of copolymers with different POSS contents characterized by average molecular weights in the range of 42,000–200,000 Da and PDI values in the range of 1.3–1.9. The surface morphology and thermal properties of hybrids were found to be affected by the POSS macromer. TEM analysis of copolymer films revealed the presence of POSS agglomerates. An analogous macromer bearing POSS-bound via phenylene linker was used in the synthesis of a series of polymers and copolymers with 3-(trifluoromethyl)phenyl-7-oxanorbornene-5,6-dicarboximide (Scheme 25) [48]. It was found that the increase in the content of POSS units in the copolymer results in a decrease in thermal stability and *T*_g_ values. TEM and AFM microimages show spherical POSS aggregates uniformly dispersed within the copolymer. POSS-substituted polynorbornenes, in which POSS groups are linked to the polynorbornene backbone through the flexible spacer with different lengths, were subjected to homopolymerization by ROMP and copolymerization with norbornene substituted with a butyl ester group, to determine the effect of the spacer length on POSS crystallization ability and the composition dependence of physical properties of the copolymers [49]. A series of homopolymers and random copolymers were synthesized in the presence of third generation Grubbs catalyst **Ru-6** in CH_2_Cl_2_, at room temperature (Figure 6) [49].

It has been demonstrated that the length of the spacer affects the crystallizability of POSS groups so that the use of a reasonably long spacer to link the POSS groups to the main chain can make POSS groups crystallizable.

**Scheme 26:**
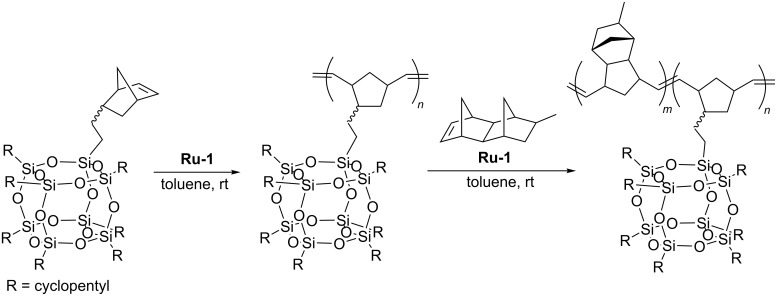
Ring-opening metathesis copolymerization of POSS-NBE with methyltetracyclododecene.

Kim and Kwon have shown that ring-opening metathesis copolymerization of norbornenylethyl-POSS with methyltetracyclododecene in the presence of first generation Grubbs catalyst (**Ru-1**) is a practical route to the synthesis of block copolymers containing POSS nanoparticles (Scheme 26) [50]. ROMP of norbornenylethyl-POSS produced the corresponding homopolymer in relatively controlled molecular weights (*M*_n_ = 17,900–26,300 Da) and narrow molecular weight distributions (in the range *M*_n_/*M*_w_ = 1.19–1.29). Copolymerization was employed by a sequential monomer addition. At first, the POSS-NBE was introduced into the reaction system containing the catalyst and after its complete conversion methyltetracyclododecene was added. The reaction was terminated with ethyl vinyl ether as soon as the second monomer was fully converted. A series of copolymers with different POSS-NBE content were obtained. The PDI values were in the range of 1.32–1.53 with average molecular weights of ca. 48000–63000 Da.

**Scheme 27:**
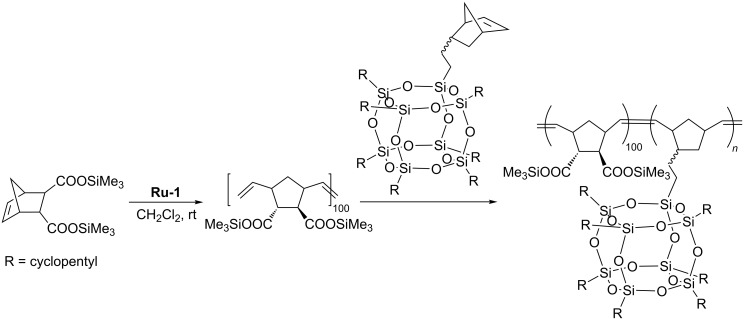
Synthesis of block copolymer via ROMP by sequential monomer addition.

The synthesized POSS containing nanocomposites displayed significant improvements in their thermal stability relative to that of the polynorbornenes formed in the absence of POSS cages. Xu has reported an example of the synthesis of POSS-containing block copolymers via “living” ROMP [51]. Copolymerization of norbornenylethyloctasilsesquioxane with 2-*endo*-3-*exo*-5-norbornene-2,3-dicaboxylic acid trimethylsilyl ester was performed in the presence of **Ru-1**. The block copolymer was obtained via sequential monomer addition (Scheme 27). After hydrolysis of the ester function, the polymer was isolated by precipitation.

As a result two block copolymers were obtained. The one containing 5% of POSS units was characterized by *M*_n_ = 26200 Da and PDI = 1.16 and the other one bearing 10% of POSS-substituted monomeric units, has a number average molecular weight *M*_n_ = 33200 Da and a polydispersity index PDI = 1.23.

**Scheme 28:**
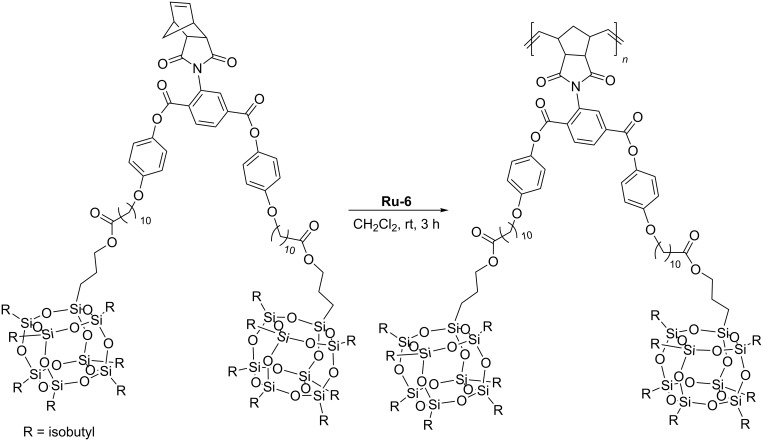
Synthesis of a liquid crystalline polymer with POSS core in the side chain.

The possibility of employing ROMP as a key step in the synthesis of a polynorbornene-based mesogen-jacketed liquid crystalline polymer (MJLCP) containing polyhedral oligomeric silsesquioxane (POSS) in the side chain was demonstrated by Shen and Fan (Scheme 28) [52]. The reaction was performed in the presence of third generation Grubbs catalyst **Ru-6** under inert atmosphere. The synthesized polymer showed various phase structures including POSS crystal and a hexagonal columnar phase, which, depending on temperature, can coexist with each other. The POSS crystal was shown to have a tremendous effect on the liquid crystalline behavior of the polymer.

**Scheme 29:**
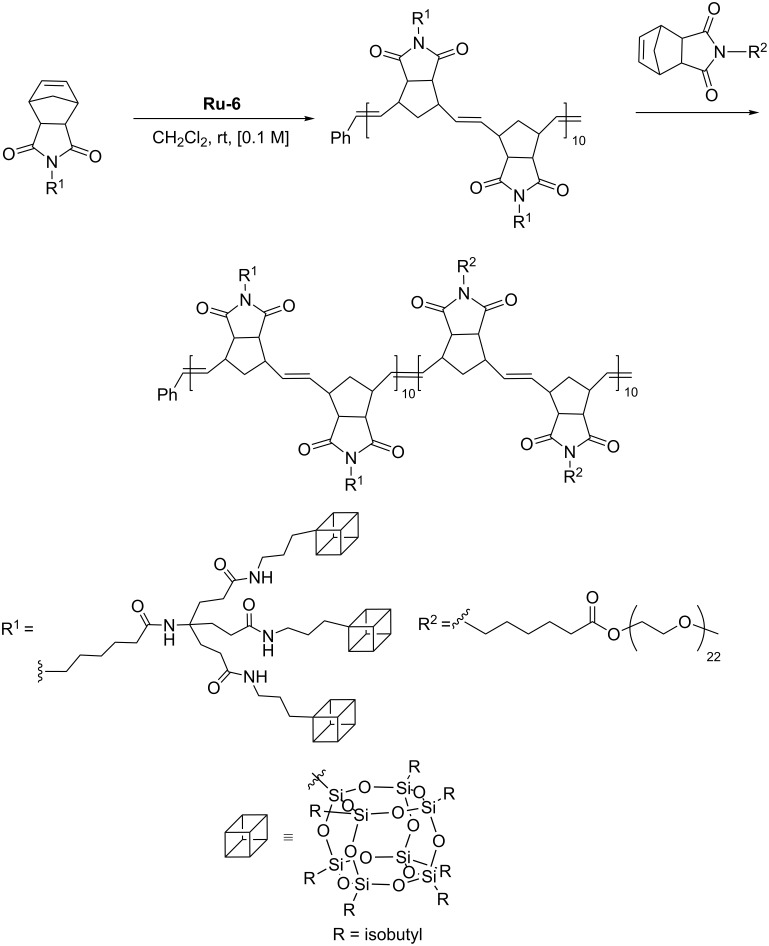
Sequential synthesis of copolymers of polynorbornene containing POSS and PEO pendant groups.

Wang has reported living ROMP of a series of monomers bearing a polymerizable norbornene dicarboxyimide group attached via an appropriate linker to 1–4 POSS units [53]. Copolymerization of POSS-bearing monomers with norbornene containing pendant poly(ethylene oxide) group permitted the synthesis of a number of block copolymers, containing blocks of hydrophobic nature (POSS containing block) and those of hydrophilic nature (polyether containing block, Scheme 29). The block copolymer was synthesized via sequential monomer addition starting from the POSS-containing macromer. The synthesis of the copolymers was carried out under mild reaction conditions in the presence of **Ru-6**. It was shown that the polymers obtained can self-assemble in THF solution into aggregates, when water was added.

**Scheme 30:**
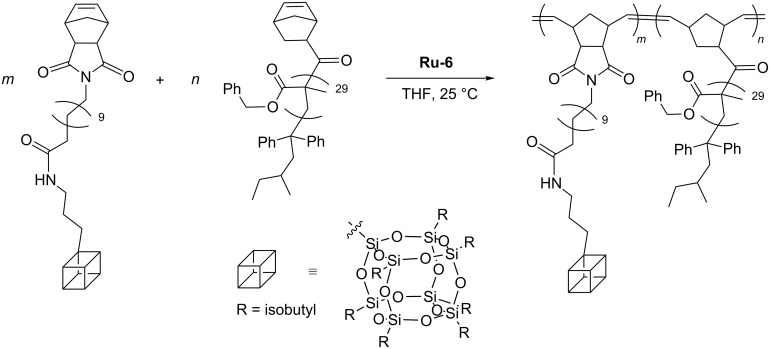
Synthesis of rodlike POSS−bottlebrush block copolymers [54].

Lee has performed a series of sequential ring-opening metathesis copolymerization of norbornene-*exo*-2,3-dicarboximido)dodecanoylamino)propylheptaisobutyl-POSS and *exo*-5-norbornene-2-carbonyl-end poly(benzyl methacrylate, Scheme 30) [54] and obtained rodlike POSS−bottlebrush block copolymers containing crystalline POSS pendants in one block and amorphous polymeric grafts in another block. Hierarchical self-assembly of rodlike copolymer was studied from the point of view of its utility in producing highly ordered 1D photonic crystals.

**Scheme 31:**
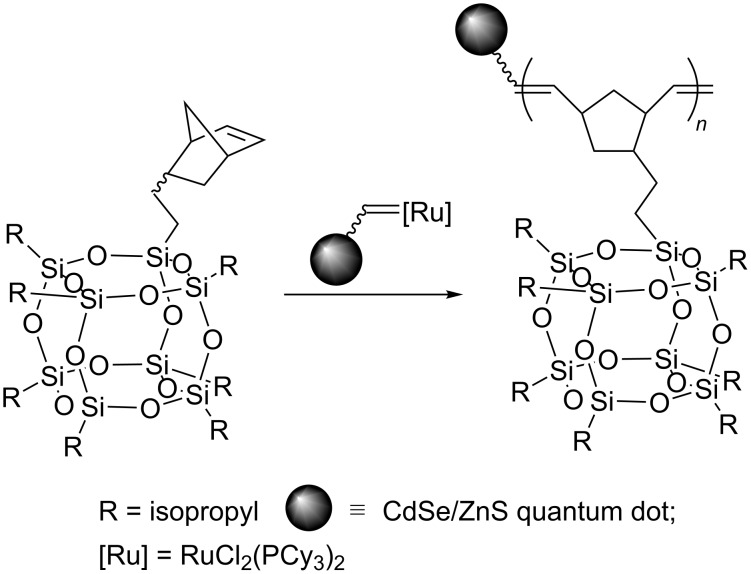
Surface-initiated ROMP producing copolymer layers on the surface of CdSe/ZnS quantum dots.

Surface-initiated ROMP was used to grow an organic corona phase on the surface of CdSe/ZnS quantum dots [39]. Functionalization of the surface with the octenyldimethylsilyl group allowed the attachment of a ruthenium alkylidene complex as a catalyst. Subsequent ROMP of norbornenylethylisobutyl cubic silsesquioxane or norbornenedicarbonyl chloride produced different molecular weights and narrow polydispersity homo- or copolymer layers directly onto the quantum dots (Scheme 31) [55].

## Conclusion

Olefin metathesis, a universal tool in organic and polymer synthesis, offers numerous advantages for the synthesis of POSS-based materials. Ruthenium-based olefin metathesis catalysts tolerate the presence of water, air and nearly all functional groups. Commercially available vinylsilsesquioxanes can be easily modified and/or functionalized by cross metathesis. According to the CM product-selectivity model [56], vinylsilsesquioxane is an olefin type III (it does not undergo homodimerization). The correct choice of olefin and catalyst permits selective CM. Another metathetic transformation – acyclic diene metathesis copolymerization – permits introduction of a POSS group to the copolymer main chain. This methodology has not been thoroughly studied so far. In turn, ring-opening metathesis (co)polymerization is a convenient tool for introducing a number of functional groups, including POSS, in the side chain of polymers. This method is limited by the small number of monomers susceptible to ROMP. In view of the dynamic development of the studies on synthesis and properties of inorganic–organic hybrid materials, it is reasonable to expect that olefin metathesis thanks to its advantages and charm will find numerous further applications in the synthesis of POSS-based materials.
